# WWP2-WWP1 Ubiquitin Ligase Complex Coordinated by PPM1G Maintains the Balance between Cellular p73 and ΔNp73 Levels

**DOI:** 10.1128/MCB.00101-14

**Published:** 2014-10

**Authors:** Neelam Chaudhary, Subbareddy Maddika

**Affiliations:** Laboratory of Cell Death and Cell Survival, Centre for DNA Fingerprinting and Diagnostics, Nampally, Hyderabad, India

## Abstract

The balance between transcription factor p73 and its functionally opposing N-terminally truncated ΔNp73 isoform is critical for cell survival, but the precise mechanism that regulates their levels is not clear. In our study, we identified WWP2, an E3 ligase, as a novel p73-associated protein that ubiquitinates and degrades p73. In contrast, WWP2 heterodimerizes with another E3 ligase, WWP1, which specifically ubiquitinates and degrades ΔNp73. Further, we identified phosphatase PPM1G as a functional switch that controls the balance between monomeric WWP2 and a WWP2/WWP1 heterodimeric state in the cell. During cellular stress, WWP2 is inactivated, leading to upregulation of p73, whereas WWP2-WWP1 complex is intact to degrade ΔNp73, thus playing an important role in shifting the balance between p73 and ΔNp73. Collectively, our results reveal a new functional E3 ligase complex controlled by PPM1G that differentially regulates cellular p73 and ΔNp73.

## INTRODUCTION

The p73 protein, also known as tumor protein 73 (TP73), belongs to the p53 family of transcription factors and has been classified as an important tumor suppressor (1). p73 is able to bind canonical p53 DNA binding sites and transactivate p53 target genes that participate in cell cycle arrest and apoptosis (2–4). p73 shares a similar domain organization as p53, including an N-terminal transactivation domain, a central DNA binding domain, and a C-terminal oligomerization domain (5). Similar to p53, several lines of evidence suggest that p73 plays an important role in human cancers. For instance, the p73 gene has been mapped to chromosome region 1p36.2-3, a locus that is frequently lost in several human cancers (6–9). Altered expression of p73 has been reported in different cancers such as neuroblastoma, breast cancers, and renal cell carcinoma (1, 10, 11). In addition, isoform-specific p73 null mice exhibit genomic instability associated with enhanced aneuploidy, which accounts for increased incidence of spontaneous tumors and carcinogen-induced tumors (12).

p73 exists in several N-terminal and C-terminal isoforms (13). The p73 gene expresses at least seven alternatively spliced C-terminal isoforms (α, β, γ, δ, ε, ζ, and η) and three alternatively spliced N-terminal isoforms. Importantly, the p73 gene can be transcribed from an alternative promoter located in intron 3 that leads to the expression of ΔNp73, an N-terminally truncated isoform. ΔNp73 lacks a transactivation domain in its structure and thereby exerts a dominant negative effect on p73 functions (14, 15). The dominant negative function of ΔNp73 may be attributed to either its ability to compete for p73 target DNA binding sites or inhibition of p73 transcriptional activity via hetero-oligomerization.

Since expression of p73 induces cell death whereas ΔNp73 protects cells from p73 induced apoptosis (16), the degree of expression of transcriptionally active p73 and inactive ΔNp73 variants determines cell fate. Thus, the fine balance between the two isoforms needs to be tightly regulated in cells. Under normal conditions, p73 and ΔNp73 levels are kept in balance (17), but upon genotoxic stress the ratio of p73 and ΔNp73 is altered in favor of p73 (2, 18). However, the precise mechanism that regulates the p73/ΔNp73 ratio under normal conditions and upon stress is not clearly understood.

WWP2 is an E3 ubiquitin ligase that plays an important role in different cellular functions such as transcription, embryonic stem cell fate, cellular transport, T-cell activation, and apoptosis (19–22). Recently, we identified WWP2 as an E3 ubiquitin ligase for PTEN (23). Although WWP2 has been identified as a potential oncogene that requires its E3 ligase activity, so far only limited substrates such as PTEN and SMADs have been implicated as functional substrates of WWP2 oncogenic function (23, 24). In addition, our previous study has shown that WWP2 negatively regulates cell death that is partially dependent on PTEN; thus, we speculated that there might be additional substrates of WWP2 in cells. In this study, we identified p73 as a novel substrate of WWP2 that might be functionally important in WWP2 prooncogenic function.

## MATERIALS AND METHODS

### Plasmids.

Full-length p73, ΔNp73, WWP1, WWP2, HACE1, E6AP, and PPM1G were cloned into a mammalian destination vector expressing an S-protein/Flag/streptavidin binding protein (SBP) triple-epitope tag (SFB) and a Myc-tagged destination vector using a Gateway cloning system (Invitrogen). Hemagglutinin (HA)-p73 and HA-ΔNp73 were also generated. p73 domain deletions were cloned into a destination vector with an S-protein/Flag/SBP triple-epitope tag. Bacterially expressing glutathione *S*-transferase (GST)–p73, GST-ΔNp73, GST-PPM1G, and maltose binding protein (MBP)-WWP2 vectors were generated by transferring their coding sequences into destination vectors. Catalytically inactive mutants of WWP1 (C890S), WWP2 (C838A), and PPM1G (D496A) were generated using a site-directed mutagenesis kit (Stratagene) according to the manufacturer's protocol. Flag-p73 and Flag-ΔNp73 were a kind gift from Alex Zaika (Vanderbilt University).

### Antibodies.

Anti-WWP2, anti-PPM1G, anti-HA (all from Bethyl Laboratories), anti-WWP1 (Abnova), antiubiquitin (anti-Ub) (Millipore), anti-MBP (New England Bio Labs), anti-Myc clone 9E10 (Santa Cruz Biotechnology), anti-Flag, anti-GST, antiactin, and antitubulin (Sigma) antibodies were used in this study.

### Tandem affinity purification.

WWP2-associated proteins were isolated by using tandem affinity purification as described previously (25). Briefly, 293T cells were transfected with WWP2 triple tagged with S-protein/Flag/SBP, and then 3 weeks later puromycin-resistant colonies were selected and screened for WWP2 expression. The WWP2-positive stable cells were then maintained in RPMI medium supplemented with fetal bovine serum (FBS) and 2 μg/ml puromycin. The SFB-WWP2 stable cells were lysed with NETN buffer (20 mM Tris-HCl, pH 8.0, 100 mM NaCl, 1 mM EDTA, 0.5% Nonidet P-40) containing 50 mM β-glycerophosphate, 10 mM NaF, and 1 μg/ml each of pepstatin A and aprotinin on ice for 30 min. After removal of cell debris by centrifugation, crude cell lysates were incubated with streptavidin-Sepharose beads (Amersham Biosciences) for 1 h at 4°C. The bound proteins were washed three times with NETN buffer and then eluted twice with 2 mg/ml biotin (Sigma) for 60 min at 4°C. The eluates were incubated with S-protein–agarose beads (Novagen) for 1 h at 4°C and then washed three times with NETN buffer. The proteins bound to S-protein–agarose beads were resolved by SDS-PAGE and visualized by Coomassie blue staining. The identities of eluted proteins were revealed by mass spectrometry analysis performed by the Taplin Biological Mass Spectrometry Facility at Harvard.

### Cell transfections, immunoprecipitation, and immunoblotting.

HEK293T and HeLa cells were transfected with various plasmids using Lipofectamine (Invitrogen) according to the manufacturer's protocol and treated accordingly. For immunoprecipitation assays, cells were lysed with NETN buffer containing 1 μg/ml each of pepstatin A, aprotinin, and phenylmethylsulfonyl fluoride (PMSF) on ice for 30 min. The whole-cell lysates obtained by centrifugation were incubated with Flag-agarose or streptavidin-Sepharose beads (Amersham Biosciences) for 1 h at 4°C. The immunocomplexes were then washed with NETN buffer four times and applied to SDS-PAGE. Immunoblotting was performed according to standard protocols.

### *In vitro* binding assays.

Bacterially expressed GST-p73, GST-ΔNp73, or control GST bound to glutathione-Sepharose beads (Amersham) was incubated with bacterially purified MBP-WWP2 or MBP-WWP1 for 1 h at 4°C, and the washed complexes were eluted by boiling in SDS sample buffer and separated by SDS-PAGE, and the interactions were analyzed by Western blotting. A similar binding assay was performed to analyze PPM1G interaction with cellular WWP2.

### Cycloheximide chase assay.

HEK293T cells were transfected with various combinations of plasmids, and at 24 h posttransfection cycloheximide (50 μg/ml) was added. Cells were harvested at different time points, and protein levels were determined by immunoblotting.

### *In vivo* ubiquitination assay.

Cells were transfected with various combinations of plasmids. At 24 h posttransfection, cells were treated with MG132 (10 μM) for 6 h, and whole-cell extracts were prepared by NETN lysis were subjected to immunoprecipitation of the substrate protein. The analysis of ubiquitination was carried out by immunoblotting with antiubiquitin antibodies.

### *In vitro* ubiquitination assay.

The reactions were carried out at 30°C for 15 min in 25 μl of ubiquitylation reaction buffer (40 mM Tris-HCl at pH 7.6, 2 mM dithiothreitol [DTT], 5 mM MgCl_2_, 0.1 M NaCl, 2 mM ATP) containing the following components: 100 μM ubiquitin, 20 nM E1 (UBE1), and 100 nM UbcH5b (all from Boston Biochem); bacterially purified MBP-WWP2 and MBP-WWP1 E3 ligases were added to the reaction mixture. Bacterially purified GST, GST-p73, and GST-ΔNp73 bound to glutathione-Sepharose beads (Amersham) were used as substrates in the reaction mixture. After the reaction, beads were washed three times with NETN buffer and boiled with SDS-PAGE loading buffer; ubiquitination of substrates was detected by Western blotting with anti-GST antibody.

### Gel filtration.

293T cell lysates (0.8 ml) were fractionated using a Sephacryl S-100 column (GE Healthcare). Fraction sizes of 500 μl were collected using a Bio-Rad 2110 fraction collector. Samples were analyzed by immunoblotting after SDS-PAGE separation.

### Pulse-chase analysis.

293T cells transfected with various combinations of plasmids/small interfering RNAs (siRNAs) were starved for 1 h in Dulbecco's modified Eagle's medium (DMEM) with dialyzed serum and then labeled with 200 μCi/ml of [^35^S]Met-Cys. Unlabeled Met and Cys (2 mM) were added, and cells were collected at the indicated time points (see Fig. 2g, 4f, and 7e). Cells were lysed with NETN buffer containing 1 μg/ml each of pepstatin A, aprotinin, and PMSF on ice for 30 min. The whole-cell lysates obtained by centrifugation were incubated with Flag-agarose for 1 h at 4°C. Immunoprecipitates were washed three times in NETN buffer and run on an SDS gel, followed by detection with autoradiography.

### RNA interference.

Vectors containing a control short hairpin RNA (shRNA) and WWP2 shRNA (shRNA1, 5′-CAGGAUGGGAGAUGAAAUAUU-3′; shRNA2, 5′-ACAUGGAGAUACUGGGCAAUU-3′), WWP1 shRNA (shRNA1, 5′-ATTGCTTATGAACGCGGCT-3′; shRNA 2, ACAACACACCTTCATCTCC-3′) were purchased from Open Biosystems and were transfected into cells with Lipofectamine using standard protocols. WWP2 siRNA described earlier (23) and prevalidated siRNAs for PPM1G (catalog numbers S102658684 and S102658691) were purchased from Qiagen and transfected using Oligofectamine using standard protocols.

### Apoptosis assays.

Cells were washed with phosphate-buffered saline (PBS) and then treated with propidium iodide hypotonic lysis buffer (0.1% sodium citrate, 0.1% Triton X, 100 μg/ml RNase, 50 μg/ml propidium iodide). After 30 min of incubation, the samples were analyzed by flow cytometry, and the percentage of apoptotic cells was calculated based on the sub-G_1_ peak.

## RESULTS

### p73 is a novel WWP2-associated protein.

**FIG 1:**
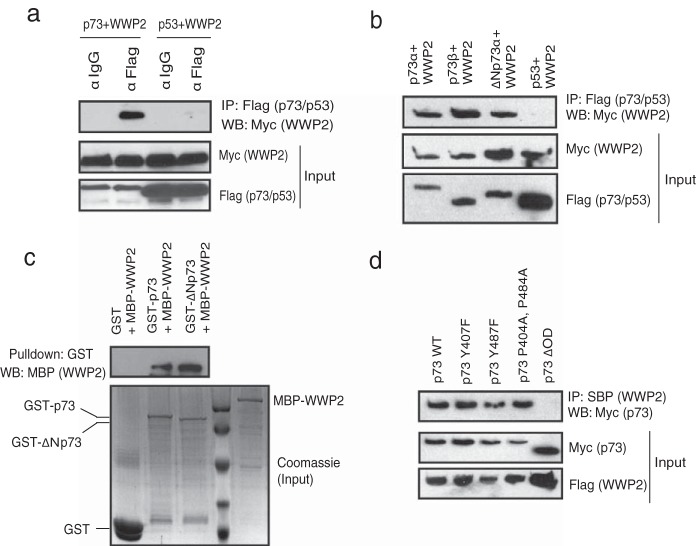
p73 is a WWP2-associated protein. (a) 293T cells were transfected with either Flag-tagged p73 or SFB-tagged p53 together with Myc-tagged WWP2. Immunoprecipitation was performed using anti-IgG or anti-Flag antibody, followed by immunoblotting with anti-Myc antibody. (b) 293T cells were separately transfected with Flag-p73α, Flag-p73β, Flag-ΔNp73α, and SFB-p53 together with Myc-WWP2. Immunoprecipitation was performed using anti-Flag followed by immunoblotting with anti-Myc antibody. (c) Bacterial cell lysate expressing MBP-WWP2 was added to GST, GST-p73, or GST-ΔNp73 immobilized on agarose beads. The *in vitro* interaction of WWP2 was assessed by immunoblotting with MBP antibody. The expression of GST, GST-p73, GST-ΔNp73, and MBP-WWP2 was shown by Coomassie staining. (d) The indicated mutants of p73 were coexpressed along with WWP2, and their interaction was determined by immunoblotting with Myc antibody after immunoprecipitation using streptavidin beads (SBP). α, anti; IP, immunoprecipitation; WB, Western blotting.

In order to identify additional substrates and/or regulators of WWP2, we established a 293T cell line stably expressing WWP2 with a triple-epitope tag (S-protein, Flag, and streptavidin-binding peptide). Tandem affinity purification of WWP2 followed by mass spectrometry analysis gave us a list of several WWP2-associated proteins. p73 was found as one of the potential interacting proteins in WWP2 complex (see List S1 in the supplemental material). By performing coimmunoprecipitation experiments using cells expressing p73 and WWP2, we confirmed that WWP2 specifically interacts with p73 but not with its closely related protein p53 (Fig. 1a). As p73 exists in several isoforms, we then tested if WWP2 interaction with p73 is specific to its isoforms. Coimmunoprecipitation experiments revealed that WWP2 interacts with both p73α and p73β. Interestingly, WWP2 also interacts with ΔNp73α, an alternate isoform and a known negative regulator of full-length p73 (Fig. 1b). Since p73α and ΔNp73α are functionally antagonistic, all of our further experiments are focused on understanding the significance of interaction between WWP2 and these two p73 isoforms. Our *in vitro* binding experiments show that a bacterially purified WWP2 interacts with bacterially expressed GST-p73 and GST-ΔNp73 but not GST alone, thus suggesting a direct interaction between WWP2 and p73 isoforms (Fig. 1c). Previous studies have shown that p73 associates with its E3 ligases through its PPXY motifs (17). As WWP2 contains WW domains, which have high affinity toward PPXY motifs, we further tested if they interact through these regions. By using various deletion mutants of p73 (see Fig. S1a in the supplemental material), we showed that it interacts with WWP2 through its oligomerization domain (see Fig. S1b) but not the region spanning PPXY motifs, suggesting that these motifs are dispensable for their interaction. In fact, mutations in either or both PPXY motifs of p73 have no effect on the interaction of PPXY with WWP2, but deletion of the oligomerization domain in p73 (p73ΔOD) abolishes their interaction (Fig. 1d). On the other hand, deletion analysis of WWP2 revealed that the WW3 domain is required for its interaction with p73 (see Fig. S1c and d). Thus, WWP2 interacts with p73 in a PPXY-independent manner via its WW domain.

### WWP2 differentially regulates stability of p73 and ΔNp73.

**FIG 2:**
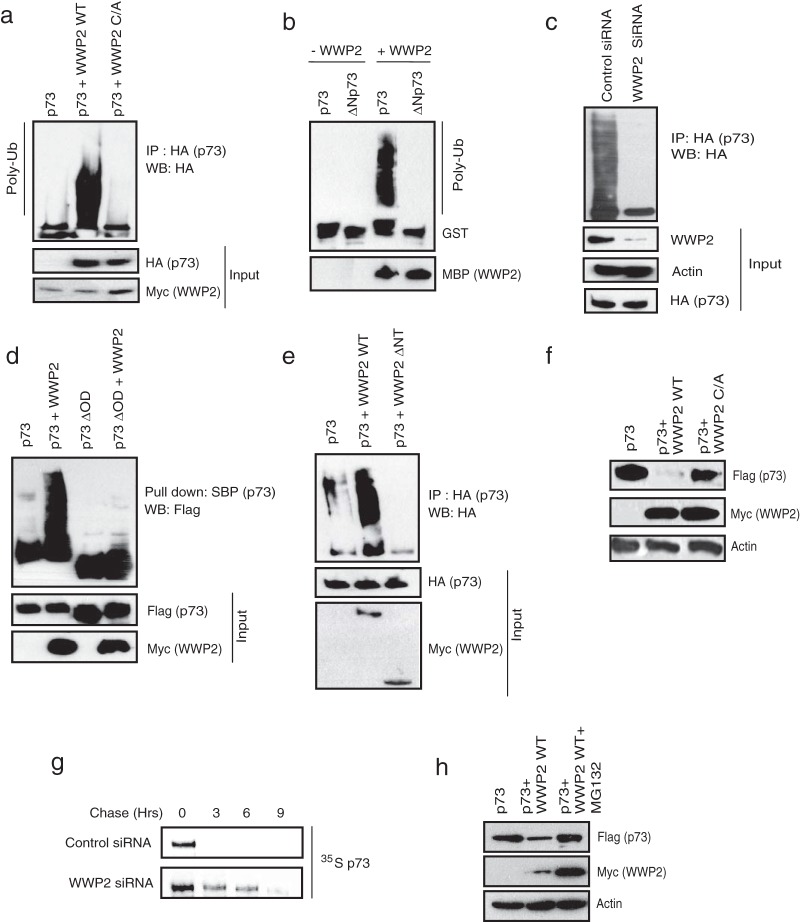
WWP2 ubiquitinates p73 and controls its protein stability. (a) 293T cells were transfected with HA-p73 alone, p73 with the wild-type (WT) WWP2, or p73 with a catalytically inactive WWP2 C838A (C/A) mutant. At 24 h posttransfection, cells were treated with MG132 for 6 h, and p73 ubiquitination was detected by immunoblotting with HA antibody after immunoprecipitation with anti-HA antibody. (b) *In vitro* ubiquitination experiments were performed using GST-p73 and GST-ΔNp73 as substrates in the presence and absence of MBP-tagged WWP2 along with E1 (UBE1) and E2 (UbcH5B) (Poly-Ub). Ubiquitinated species of GST-p73 and GST-ΔNp73 were detected by immunoblotting with anti-GST antibody. (c) HeLa cells were transfected with control siRNA or WWP2 siRNA. After 24 h of transfection, cells were transfected with HA-p73 construct, and 18 h later cells were treated with MG132 (10 μM) for 6 h before cell lysates were collected. Immunoprecipitation was done using anti-HA followed by immunoblotting with HA antibody. (d) 293T cells were transfected with SFB-tagged p73 or p73ΔOD with or without WWP2. At 24 h posttransfection cells were treated with MG132 for 6 h, and ubiquitination was detected by immunoblotting with Flag antibody after pulldown with SBP beads. (e) 293T cells were transfected with p73 alone, p73 with WWP2 wild type (WT), or p73 with WWP2 with an N-terminal deletion (ΔNT). At 24 h posttransfection, cells were treated with MG132 for 6 h, and ubiquitination was detected by immunoblotting with HA antibody after immunoprecipitation with anti-HA antibody. (f) Cells were transfected with either SFB-p73 alone, p73 with Myc-WWP2 wild type (WT), or p73 with a WWP2 C838A (C/A) mutant. Levels of p73 were detected by immunoblotting with anti-Flag antibody. (g) 293T cells were transfected with control siRNA or WWP2 siRNA. At 24 h posttransfection, cells were transfected with Flag-tagged p73. After 36 h of transfection, cells were labeled with 200 μCi/ml of ^35^S-labeled Met-Cys. Unlabeled Met and Cys (2 mM) were added, and cells were collected at indicated time points. Immunoprecipitation was performed using anti-Flag antibody, and protein levels were detected by autoradiography. (h) 293T cells were transfected with the indicated plasmids, and levels of p73 in the absence and presence of WWP2 wild type with and without MG132 treatment were determined using anti-Flag antibody.

Since WWP2 is a known HECT-type E3 ligase that regulates the functions of its substrates by ubiquitination, we further tested whether p73 is a functional substrate of WWP2. Our *in vivo* ubiquitination assays revealed that the wild-type but not a catalytically inactive C838A mutant of WWP2 promotes ubiquitination of p73 in cells (Fig. 2a; see also Fig. S2a in the supplemental material). Further, by using an *in vitro* ubiquitin conjugation assay, we showed that a bacterially purified WWP2 actively ubiquitinated recombinant p73 (Fig. 2b). On the other hand, knockdown of WWP2 dramatically reduced the levels of p73 ubiquitination, clearly suggesting that WWP2 acts as an E3 ligase for p73 (Fig. 2c; see also Fig. S2b). Full-length p73, but not its oligomerization domain deletion mutant (p73ΔOD) that is defective in interaction with WWP2, is readily ubiquitinated by WWP2 (Fig. 2d). Conversely, deletion of the N-terminal region of WWP2 that is required for its interaction with p73 severely abolished its ability to ubiquitinate p73 (Fig. 2e), thus suggesting that the interaction of WWP2 is critical for p73 ubiquitination. WWP2 mediates p73 ubiquitination via K48 linkage as a mutant ubiquitin with all lysines except lysine 48 replaced with arginine is sufficient to form polyubiquitin chains on p73 (see Fig. S2c). To further characterize the functional significance of WWP2-mediated p73 ubiquitination, we checked p73 levels in cells expressing WWP2. As shown in Fig. 2f, expression of wild-type WWP2 but not its catalytically inactive mutant downregulated cellular p73 levels. Indeed, our pulse-chase experiments suggested that depletion of WWP2 significantly increased the protein half-life of p73 (Fig. 2g). In addition, WWP2-mediated degradation of p73 is dependent on proteasome activity as its inhibition with MG132 rescued the p73 levels (Fig. 2h).

### WWP1 heterodimerizes with WWP2.

**FIG 3:**
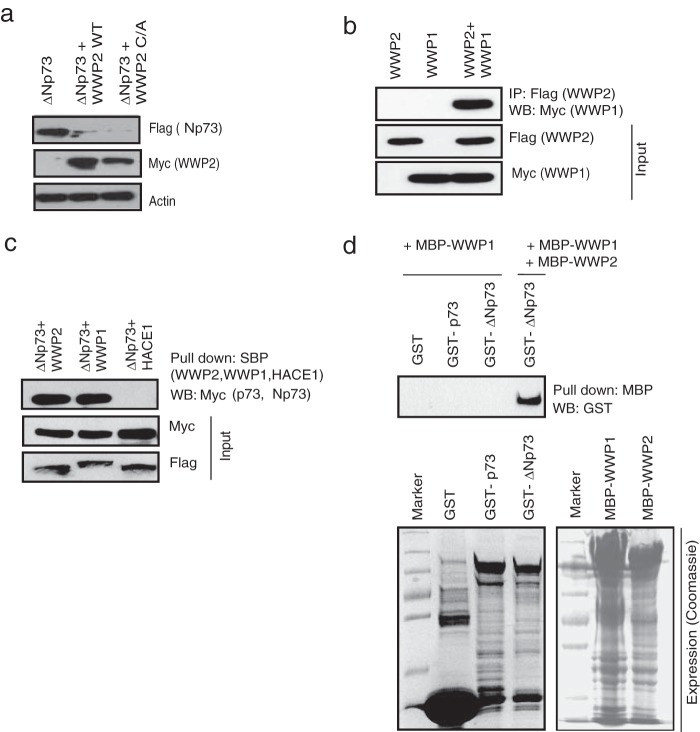
WWP1 heterodimerizes with WWP2. (a) 293T cells were transfected with the indicated plasmids, and levels of ΔNp73 in the presence of wild-type (WT) WWP2 and a WWP2 C838A (C/A) mutant were determined using anti-Flag antibody. (b) SFB-WWP2 and Myc-WWP1 alone or together were expressed in 293T cells, and their association was tested by immunoprecipitation with Flag antibody followed by immunoblotting with Myc antibody. (c) Cells were transfected with ΔNp73 in combination with WWP2, WWP1, or HACE1, and ΔNp73 interaction with the E3 ligases was determined by immunoblotting with Myc antibody after pulldown with SBP beads. (d) Bacterial cell lysate expressing GST, GST-p73, or GST-ΔNp73 was added to MBP-WWP2 and MBP-WWP1 immobilized on Sepharose beads as indicated. The *in vitro* interaction was assessed by immunoblotting with GST antibody. The expression of GST, GST-p73, GST-ΔNp73, MBP-WWP1, and MBP-WWP2 was shown by Coomassie staining.

As we found that ΔNp73 also interacts with WWP2, we further tested if WWP2 acts as an E3 ligase for this isoform as well. Interestingly, both the wild type and a C838A mutant enhanced ΔNp73 ubiquitination (see Fig. S3a and b in the supplemental material) followed by its degradation (Fig. 3a). These results suggest that WWP2 might be regulating p73 and ΔNp73 by two distinct mechanisms. Since WWP2 degrades ΔNp73 independent of its catalytic function, we hypothesized that WWP2 might be recruiting another E3 ligase for degradation of ΔNp73 specifically. To test this hypothesis, we analyzed the list of WWP2-associated proteins generated by tandem affinity purification. In fact, we found that WWP1, another HECT-type E3 ligase (26), is associated with the purified WWP2 complex (see List S1 in the supplemental material). By using coimmunoprecipitation experiments, we confirmed the heterodimerization of WWP2 and WWP1 in cells (Fig. 3b). Further, we tested whether WWP1 interacts with p73 and ΔNp73. We found that WWP1 along with WWP2 but not a control E3 ligase, HACE1, associates with ΔNp73 (Fig. 3c). Our *in vitro* binding experiments using bacterially purified recombinant proteins suggested that WWP1 specifically interacts with ΔNp73 but requires WWP2 for their association (Fig. 3d).

### WWP1 in complex with WWP2 specifically regulates ΔNp73.

**FIG 4:**
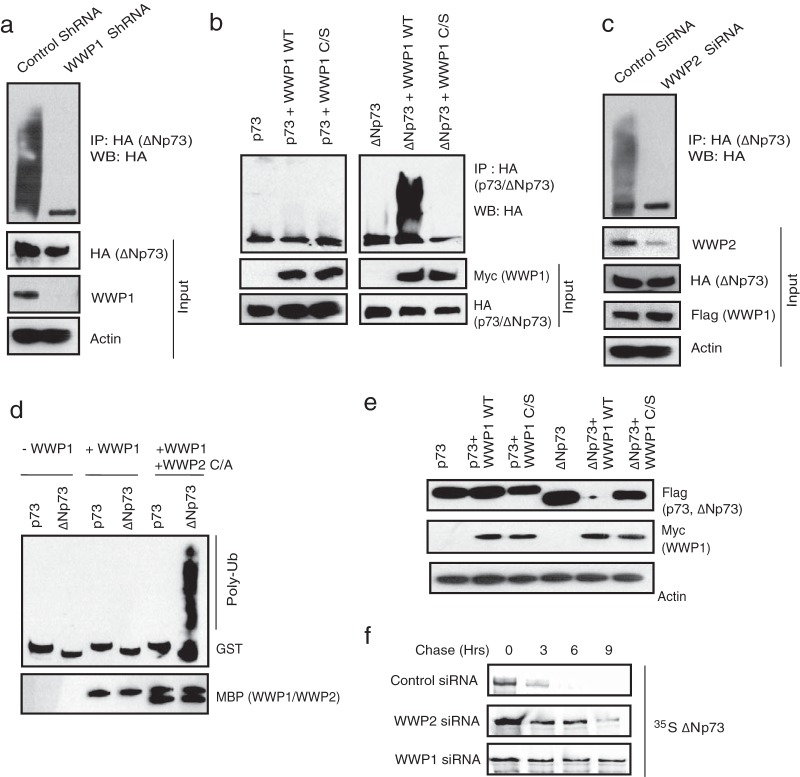
WWP1 in complex with WWP2 specifically regulates ΔNp73. (a) HeLa cells were transfected with control shRNA or WWP1 shRNA. After 48 h, HA-ΔNp73 was transfected, and cells were treated with MG132 for 6 h before harvesting. Cell lysates were subjected to immunoprecipitation, and ubiquitination of ΔNp73 was detected by immunoblotting with anti-HA antibody. (b) 293T cells were transfected as indicated, and cells were treated with MG132 for 6 h. Ubiquitination of p73 and ΔNp73 was detected by immunoblotting with HA antibody after immunoprecipitation with HA antibody. (c) HeLa cells were transfected with control siRNA or WWP2 siRNA. After 24 h of transfection, cells were transfected with HA-ΔNp73 and SFB-WWP1. After 18 h cells were treated with MG132 (10 μM). WWP1-mediated ΔNp73 ubiquitination was analyzed by immunoprecipitation using anti-HA, followed by immunoblotting with HA antibody. (d) *In vitro* ubiquitination experiments were performed using bacterially purified GST-p73 and GST-ΔNp73 as a substrate in the indicated combinations with MBP-tagged WWP1 and an MBP-tagged WWP2 C838A (C/A) mutant along with E1 (UBE1) and E2 (UbcH5B). Ubiquitinated species of GST-p73 and GST-ΔNp73 were detected by immunoblotting with anti-GST antibody. (e) 293T cells were transfected with p73 alone, ΔNp73 alone, or p73 or ΔNp73 in combination with wild-type (WT) WWP1 or with a catalytically inactive WWP1 C890S (C/S) mutant. The levels of p73 and ΔNp73 were detected by immunoblotting with anti-Flag antibody. (f) 293T cells were transfected with a control siRNA, WWP2 siRNA, and WWP1 siRNA. At 24 h after siRNA transfection, cells were transfected with Flag-tagged ΔNp73. After 36 h of transfection, cells were labeled with 200 μCi/ml of ^35^S-labeled Met-Cys. Unlabeled Met and Cys (2 mM) were added, and cells were collected at the indicated time points. Immunoprecipitation was performed using anti-Flag antibody, and levels were detected by autoradiography.

**FIG 5:**
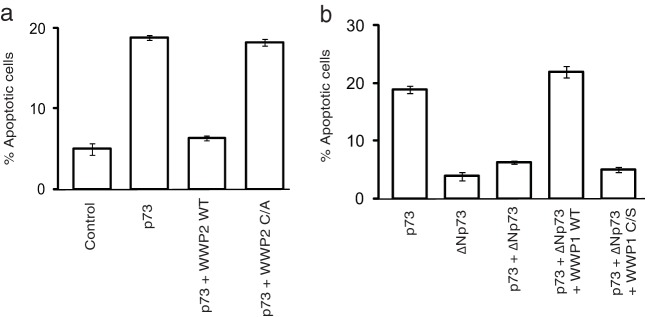
WWP2-WWP1 complex controls p73-induced apoptosis. (a) HeLa cells were transfected with a vector control, p73 alone, or p73 in combination with wild-type WWP2 (WT) and a WWP2 C838A (C/A) mutant. Cells were stained with propidium iodide in hypotonic buffer, and the percentage of apoptosis was determined by sub-G_1_ peak analysis by using flow cytometry. Error bars indicate standard deviations (*n* = 3; *P* < 0.01; Student's *t* test). (b) HeLa cells were transfected with various constructs as indicated, and the percentage of apoptosis was determined by propidium iodide staining followed by sub-G_1_ peak analysis using flow cytometry. Error bars indicate standard deviations (*n* = 3; *P* < 0.01; Student's *t* test).

Since WWP1 is also a bona fide E3 ligase, we then tested if it is required for ΔNp73 ubiquitination. Knockdown of WWP1 by shRNA significantly reduced the polyubiquitinated species of ΔNp73 in cells (Fig. 4a). On the other hand, WWP1 has no effect on full-length p73 ubiquitination but is able to readily promote ubiquitination of ΔNp73 (Fig. 4b). WWP1-mediated ubiquitination of ΔNp73 is dependent on its catalytic activity as a C890S mutant was inefficient in promoting ΔNp73 ubiquitination, thus confirming the role of WWP1 as a specific E3 ligase for ΔNp73. Interestingly, depletion of WWP2 reduced the ability of WWP1 to polyubiquitinate ΔNp73 (Fig. 4c); thus, assembly of a heterodimeric complex of WWP2-WWP1 is essential for efficient ΔNp73 polyubiquitination. Further, by using an *in vitro* ubiquitin conjugation assay, we confirmed that ΔNp73 is specifically ubiquitinated by WWP1-WWP2 E3 ligase complex (Fig. 4d). Since we observed that ΔNp73 is ubiquitinated by K48 linkages (see Fig. S3c in the supplemental material), we next tested if polyubiquitination of ΔNp73 by WWP1 leads to its degradation. Expression of wild-type WWP1 but not the C890S mutant reduced the levels of ΔNp73 (Fig. 4e). Of note, WWP1 expression has no effect on full-length p73 levels, again supporting the distinct role of WWP2-WWP1 complex in controlling ΔNp73 specifically. Our pulse-chase experiments further confirmed that depletion of either WWP2 or WWP1 significantly increased the half-life of ΔNp73 (Fig. 4f). Thus, we found that WWP1 was a WWP2-associated E3 ligase for ΔNp73. Further, to understand the functional significance of the interaction of p73 isoforms with WWP2 and WWP1, we tested the effect of these E3 ligases on the apoptosis-controlling ability of p73 and ΔNp73. Expression of p73 readily induces apoptosis, which is rescued by cotransfection with wild-type WWP2 but not a catalytically inactive mutant (Fig. 5a). On the other hand, ΔNp73 suppresses p73-mediated apoptosis, which is relieved by coexpression of its E3 ligase WWP1 (Fig. 5b). The relieving effect of WWP1 on ΔNp73-mediated inhibition of p73 is again dependent on its catalytic activity as the C890S mutant could not relieve the inhibition. These results suggest that WWP2 might exist in two functionally distinct forms, wherein WWP2 in monomeric form ubiquitinates and degrades full-length p73 but in a heterodimeric complex with WWP1 regulates ΔNp73, thus maintaining a fine balance between these two isoforms in cells.

### PPM1G inactivates monomeric WWP2 and promotes the assembly of WWP2-WWP1 heterodimeric complex.

**FIG 6:**
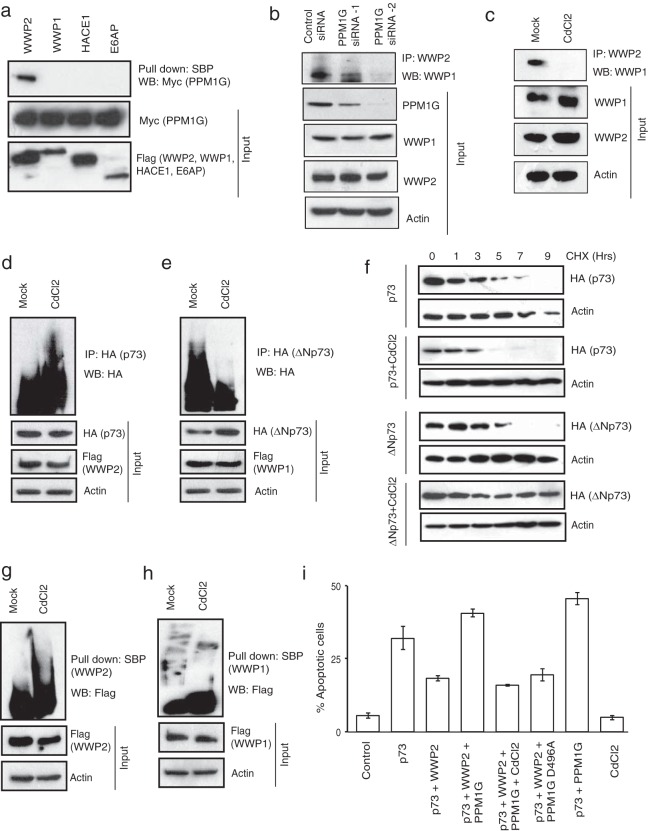
PPM1G inactivates monomeric WWP2 and promotes the assembly of WWP2-WWP1 heterodimeric complex. (a) SFB-tagged WWP2, WWP1, HACE1, and E6AP along with Myc-PPM1G were expressed in 293T cells, and their association was tested by pulldown with SBP beads, followed by immunoblotting with anti-Myc antibody. (b) HeLa cells were transfected with control siRNA or two individual PPM1G siRNAs. The presence of WWP2-WWP1 heterodimeric complex in these cells was analyzed by immunoprecipitation with WWP2 antibody followed by immunoblotting with WWP1 antibody. (c) Cells were either mock treated with buffer or treated with CdCl_2_ (1.5 μM), and the association of WWP2-WWP1 in these cells was analyzed by immunoprecipitation with WWP2 antibody followed by immunoblotting with WWP1 antibody. (d) 293T cells were transfected with p73 and WWP2, and 24 h later they were either mock treated or treated with CdCl_2_. WWP2-mediated p73 ubiquitination was detected by immunoblotting with HA antibody after immunoprecipitation with anti-HA antibody. (e) Cells were transfected with ΔNp73 and WWP1, and 24 h later they were either mock treated or treated with CdCl_2_. WWP1-mediated ΔNp73 ubiquitination was detected by immunoblotting with HA antibody after immunoprecipitation with anti-HA antibody. (f) 293T cells transfected with p73 or ΔNp73 were either mock treated or treated with CdCl_2_. Later cells were treated with cycloheximide (CHX) and were collected at the indicated time points. Levels of p73 and ΔNp73 were determined by immunoblotting with anti-HA antibody. (g) Cells expressing SFB-WWP2 were either mock treated or treated with CdCl_2_. After 6 h of MG132 treatment, WWP2 activity was assayed by detecting its autoubiquitination levels using Flag antibody after pulldown with SBP beads. (h) Cells expressing SFB-WWP1 were either mock treated or treated with CdCl_2_. After 6 h of MG132 treatment, WWP1 activity was assayed by detecting its auto-ubiquitination levels using Flag antibody after pulldown with SBP beads. (i) HeLa cells were transfected with various constructs as indicated, and the percentage of apoptosis was determined by propidium iodide staining followed by sub-G_1_ peak analysis using flow cytometry. Error bars indicate standard deviations (*n* = 3, *P* < 0.01; Student's *t* test).

As our results suggested that the interplay between the monomeric and heterodimeric WWP2 state is critical in regulating p73 and ΔNp73 levels, we hypothesized that a molecular switch might exist in the cells that may alter WWP2 in these two states under different cellular conditions. To test this hypothesis, we again analyzed the list of WWP2-associated proteins generated by tandem affinity purification. After testing various listed WWP2-associated proteins, we found that a phosphatase, PPM1G, specifically interacts with WWP2 (Fig. 6a; see also Fig. S4a in the supplemental material). PPM1G, also known as PP2Cγ, is a Mg^2+^/Mn^2+^-dependent nuclear serine/threonine phosphatase that plays an important role in different functions, such as nucleosome assembly, cell survival control, mRNA splicing, and DNA damage response (27–29).

First, we ruled out the possibility of PPM1G as a substrate of WWP2 since we did not observe any changes in the levels of PPM1G with increasing concentrations of WWP2 (see Fig. S4b in the supplemental material). Next, to test the possibility of PPM1G as a molecular switch for monomeric versus heterodimeric WWP2, we depleted PPM1G in cells and tested for a WWP2-WWP1 interaction. As shown in Fig. 6b, depletion of PPM1G severely affected the association of WWP1 and WWP2. PPM1G activity is required for the assembly of WWP2-WWP1 complex as inhibition of PPM1G activity by cadmium chloride led to the loss of WWP2-WWP1 interaction in cells (Fig. 6c). Since PPM1G is important for the assembly of WWP2-WWP1 complex, we further tested its effect on p73 and ΔNp73. We observed that p73 ubiquitination is enhanced (Fig. 6d), whereas ΔNp73 ubiquitination is significantly reduced upon inhibition of PPM1G activity (Fig. 6e). The altered ubiquitination of p73 and ΔNp73 by PPM1G is important in regulating their protein stability as inhibition of PPM1G reduced the protein half-life of p73, whereas ΔNp73 was strongly stabilized (Fig. 6f). Our further experiments revealed that the presence of active PPM1G inhibits the E3 ligase activity of WWP2 as cadmium chloride treatment of cells led to enhanced autoubiquitination of WWP2 (Fig. 6g). On the other hand, PPM1G has no significant effect on WWP1 activity (Fig. 6h). Further, to test if PPM1G negatively regulates WWP2 function, we analyzed its effect on WWP2-controlled p73-mediated apoptosis. As expected, WWP2 reduced p73-induced cellular apoptosis, which is rescued by coexpression of PPM1G (Fig. 6i). The rescue effect of PPM1G is dependent on its catalytic activity as cadmium chloride treatment or transfection of a catalytically inactive PPM1G mutant reversed the apoptotic phenotype. In addition, depletion of PPM1G by siRNA reduced the ability of p73 to induce apoptosis (see Fig. S4c in the supplemental material). Together, these results suggest that PPM1G acts as functional molecular switch that promotes the assembly of a WWP2-WWP1 heterodimeric complex and at the same time specifically inhibits the E3 ligase activity of WWP2 and thus controls the balance between their substrates, p73 and ΔNp73.

### WWP2-WWP1 complex alters the balance between p73 and ΔNp73 during cellular stress.

**FIG 7:**
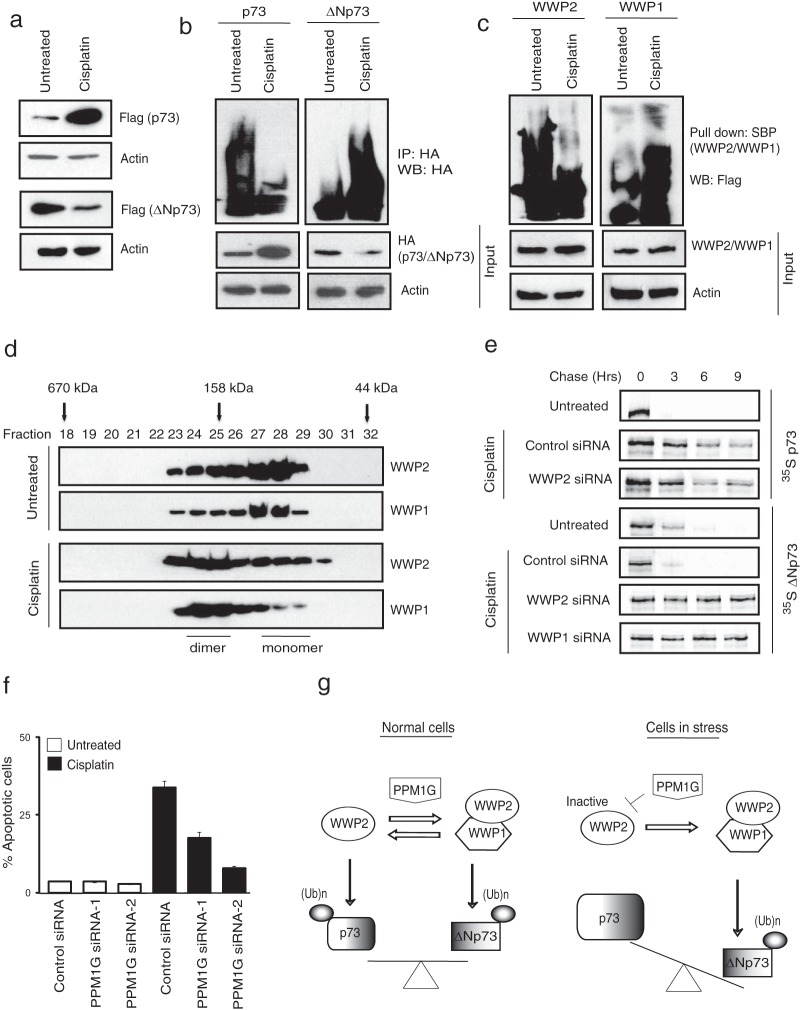
WWP2-WWP1 complex alters the balance between p73 and ΔNp73 during stress. (a) HeLa cells expressing p73 and ΔNp73 were either left untreated or treated with cisplatin (20 μM) for 24 h, and their protein levels were detected by immunoblotting with Flag antibody. (b) Cells were transfected with HA-tagged p73 and HA-tagged ΔNp73. After 24 h of transfection, cells were left untreated or treated with cisplatin. Cells were harvested after 6 h of MG132 treatment, and the levels of substrate ubiquitination were determined by immunoblotting with HA antibody after immunoprecipitation with HA antibody. (c) Cells were either left untreated or treated with cisplatin. After 6 h of MG132 treatment, activity of WWP2 and WWP1 was assayed by detecting their autoubiquitination levels using Flag antibody after pulldown with SBP beads. (d) Cell lysates from 293T cells either untreated or treated with cisplatin were fractionated through gel filtration, and the proteins were detected by Western blotting with the indicated antibodies. (e) 293T cells were transfected with a control siRNA, WWP2 siRNA, and WWP1 siRNA. At 24 h after siRNA transfection, cells were transfected with Flag-tagged p73 and Flag-tagged ΔNp73. After 24 h of transfection, cells were treated with 30 μM cisplatin for 12 h; then cells were labeled with 200 μCi/ml of ^35^S-labeled Met-Cys. Unlabeled Met and Cys (2 mM) were added, and cells were collected at the indicated time points. Immunoprecipitation was performed using anti-Flag antibody, and levels were detected by autoradiography. (f) HeLa cells were transfected with either a control siRNA or two individual PPM1G siRNAs. Forty-eight hours later cells were left untreated or treated with cisplatin, and the percentage of apoptosis was determined by propidium iodide staining, followed by sub-G_1_ peak analysis using flow cytometry. Error bars indicate standard deviations (*n* = 3, *P* < 0.01; Student's *t* test). (g) A proposed model to show the role of monomeric WWP2 and PPM1G-promoted WWP2-WWP1 heterodimeric complex in regulating p73 and ΔNp73 levels under normal and stress conditions.

It is well known that during cellular stress such as DNA damage, the balance between p73 and ΔNp73 is altered whereby the proapoptotic p73 is upregulated, and the levels of antiapoptotic ΔNp73 are downregulated (14, 18). Previous studies have shown that accumulation of p73 during cellular stress might be due to increased transcription of p73 (30). But, nonetheless other investigators have reported the role of posttranslational mechanisms such as ubiquitination to alter the ratio of p73 and ΔNp73 during cellular stress (17, 31). As we clearly observed that WWP2 and WWP1 participate in ubiquitination of p73 and ΔNp73, we further tested if there exists a mechanism that involves these E3 ligases and that can alter the balance between the two isoforms. As expected, we found that p73 was upregulated while ΔNp73 was downregulated following treatment of cells with cisplatin (Fig. 7a). We next tested whether the changes in the protein levels of p73 and ΔNp73 were due to changes in their ubiquitination levels. As shown in Fig. 7b, ubiquitination of p73 is reduced, whereas ΔNp73 ubiquitination is enhanced upon DNA damage. In contrast, we did not observe any significant changes in the protein level of WWP2, WWP1, or PPM1G upon cell stress (see Fig. S5a in the supplemental material). We then tested whether changes in p73 and ΔNp73 ubiquitination upon cellular stress are due to altered activity of the E3 ligases. In fact our auto-ubiquitination assays revealed that activity of WWP2 is dramatically reduced, whereas WWP1 activity is modestly enhanced upon cisplatin treatment (Fig. 7c). We next tested whether the balance between monomeric and heterodimeric WWP2-WWP1 changes upon cellular stress. Our gel filtration analysis revealed that under normal conditions, both WWP2 and WWP1 exist in monomeric and dimeric states. But, interestingly, upon cellular stress the balance is significantly shifted toward the heterodimeric WWP2-WWP1 state from the monomeric state. Further, the accumulation of WWP2-WWP1 complex is necessary for efficient degradation of ΔNp73 during cellular stress as depletion of either WWP2 or WWP1 significantly stabilized ΔNp73 protein levels that were degraded upon cisplatin treatment alone (Fig. 7e). As PPM1G inhibits WWP2 activity, we further tested whether PPM1G is required for stress-induced cell death. In fact, cells became severely resistant to cisplatin-induced apoptosis with either pretreatment with the PPM1G inhibitor cadmium chloride (see Fig. S5b in the supplemental material) or siRNA-mediated depletion of PPM1G (Fig. 7f). Taken together, these results indicate that inactivation of WWP2 by PPM1G during cellular stress leads to specific accumulation of p73, whereas inactivated WWP2, again with the help of PPM1G, could still heterodimerize with WWP1 to destabilize ΔNp73.

## DISCUSSION

p73 is a p53-related transcription factor that exists in full-length and N-terminally truncated ΔNp73 isoforms. The full-length p73 isoform is expressed from an upstream promoter and has a strong ability to induce cell cycle arrest/apoptosis and protect against genomic instability and is thus regarded as bona fide tumor suppressor. On the other hand, the ΔNp73 isoform lacks the N terminus transactivation domain; hence, it cannot induce apoptosis but can still oligomerize with full-length p73 to block its transcriptional activity. Due to the opposing functions of these proteins in controlling cell survival, the ratio of the p73 isoform to the ΔNp73 isoform is critical in determining cell fate under normal and genotoxic stress conditions and thus needs to be tightly regulated at both transcriptional and posttranslational levels. At the posttranslational level, ubiquitination has been shown to play a very important role in regulating the protein stability of p73 and its isoforms. Itch, a HECT domain-containing protein, was identified as the first E3 ligase to mediate p73 ubiquitination (17). Subsequently, other E3 ligases such as PIR2 (31), UFD2a (32), and FBXO45 (33) have been reported to control the stability of p73 and ΔNp73, but most of these studies were insufficient to define a precise mechanism that regulates the p73/ΔNp73 ratio both under normal conditions and upon genotoxic stress. In addition, with p73 being a very critical tumor suppressor, multiple E3 ligases may function in different cellular contexts to maintain its optimal levels in cells, as has been reported with other important players such as p53 (34).

In this work, we identified a complex of WWP2-WWP1 E3 ubiquitin ligases that function together to regulate the balance between p73 and ΔNp73. We propose that under normal conditions, WWP2 exists in two different states, where E3 ligase in a monomeric form degrades p73 but in a heterodimeric complex with WWP1 regulates ΔNp73 and thus maintains a fine balance between these two isoforms in cells. On the other hand, during genotoxic stress, monomeric WWP2 is inactivated and prevents the degradation of p73, whereas inactive WWP2 can promote ΔNp73 degradation via WWP1 complex, thus shifting the balance toward accumulation of p73. PPM1G coordinates the interplay between monomeric and heterodimeric states of WWP2 and thus controls the balance between two functionally opposing isoforms of p73 (a hypothetical model is shown in Fig. 7g). Thus, our study highlights a novel mechanism of regulation of relative levels of p73 and ΔNp73 under normal conditions and during DNA damage, and the alteration of these levels might lead to carcinogenesis.

Previously, we demonstrated that WWP2 acts as a potential oncogene by negatively regulating PTEN protein stability (23), but downregulation of PTEN alone may not fully explain the oncogenic potential of WWP2. Although subsequent studies have reiterated the oncogenic role of WWP2 (35), very few functional substrates have been identified thereafter. Hence, our current study revealing tumor suppressor p73 as an additional functional downstream substrate of WWP2 E3 ligase activity certainly provided important evidence in understanding the role of this E3 ligase as a potential oncogene. It would be interesting to further analyze if a negative correlation exists between WWP2 and p73 expression in human cancers, which would further strengthen their functional link in tumorigenesis.

On the other hand, we identified phosphatase PPM1G as a novel regulator of WWP2 E3 ligase activity. Although earlier studies have implicated this phosphatase in several important cellular functions such as nucleosome assembly, cell survival control, mRNA splicing, and DNA damage response (27–29), its role in tumorigenesis has not been reported so far. Since we determined that PPM1G positively regulates p73 levels and negatively regulates its counterpart ΔNp73 by controlling WWP2, it is tempting to speculate that PPM1G might act as a potential tumor suppressor. Our current studies are focused on unraveling the new functional substrates of PPM1G and further understanding its possible role as a tumor suppressor in human cancers.

## Supplementary Material

Supplemental material
