# Supplementary material for: WWP2-WWP1 Ubiquitin Ligase Complex Coordinated by PPM1G Maintains the Balance between Cellular p73 and ΔNp73 Levels
Source: Mol Cell Biol. 2014 Oct;34(19):3754–64. doi: 10.1128/MCB.00101-14 (PMC4187731; doi:10.1128/MCB.00101-14)
Supplement: Supplemental material [file supp_34_19_3754__index.html]

Supplemental material 

# WWP2-WWP1 Ubiquitin Ligase Complex Coordinated by PPM1G Maintains the Balance between Cellular p73 and ΔNp73 Levels

## Supplemental material

**Files in this Data Supplement:**

- Supplemental file 1 -

  Legends to Fig. S1 to S5

  PDF, 111K
- Supplemental file 2 -

  List S1 (WWP2-associated proteins identified by MS analysis)

  PDF, 72K
- Supplemental file 3 -

  Fig. S1 (Schematic representation of and transfection with SFB-p73 and Myc-WWP2), S2 (Transfection with WWP2 and p73), S3 (p73 ubiquitination), and S4 and S5 (Protein expression and apoptosis)

  PDF, 2.0M
