# Supplementary material for: WWP2-WWP1 Ubiquitin Ligase Complex Coordinated by PPM1G Maintains the Balance between Cellular p73 and ΔNp73 Levels
Source: Mol Cell Biol. 2014 Oct;34(19):3754–64. doi: 10.1128/MCB.00101-14 (PMC4187731; doi:10.1128/MCB.00101-14)
Supplement: Supplemental material [file MCB.00101-14_zmb999100602so1.pdf]

Supplementary information

WWP2-WWP1 ubiquitin ligase complex co-ordinated by PPM1G maintains the balance between cellular p73 and ΔNp73 levels

Neelam Chaudhary[1] & Subbareddy Maddika[1]*

[1]Laboratory of Cell Death & Cell Survival, Centre for DNA Fingerprinting and Diagnostics (CDFD), Nampally, Hyderabad 500001, INDIA

*To whom correspondence should be addressed.

Dr. Subbareddy Maddika

Tel: +91-40-24749353

Fax: +91-40-24749448

E-mail: msreddy@cdfd.org.in

This file contains Supplementary Figure 1- 5

Supplementary Figure legends

Supplementary Figure 1: (a) Schematic representation of N-terminal SFB-p73 and its various deletion mutants (D1 to D6). **(b)** 293T cells were transfected with SFB-p73 and its deletion constructs along with Myc-WWP2. The site of interaction was determined by pull down with SBP beads followed by immunoblotting with anti-Myc antibody. **(c)** Schematic representation of N-terminal Myc-WWP2 and its various deletion mutants (D1 to D6). **(d)** 293T cells were transfected with Myc-WWP2 and its deletion constructs along with HA-p73. The site of

interaction was determined by immunoprecipitation with HA antibody followed by immunoblotting with anti-Myc antibody.

Supplementary Figure 2: (a) 293T cells were transfected with Flag-p73, p73 with wild type (WT) WWP2 or catalytically inactive (C/A) WWP2 mutant. 24 hours post transfection cells were treated with MG132 for 6 hours and p73 ubiquitination was detected by immunoblotting with ubiquitin antibody after immunoprecipitation with anti-Flag antibody. **(b)** HeLa cells were transfected with control shRNA or two individual WWP2 shRNAs. After 24 hours of transfection cells were transfected with HA-p73 construct and 18 hours later cells were treated with MG132 (10uM) for 6hrs before collecting cell lysates. Immunoprecipitation was done using anti-HA followed by immunoblotting with ubiquitin antibody. **(c)** 293T cells were transfected with SFb-p73 along with HA-tagged ubiquitin mutants. p73 polyubiquitination was detected by denaturing immunoprecipiation with anti-Flag antibody followed by detection with anti-HA (detecting ubiquitin) antibody.

Supplementary Figure 3: (a) 293T cells were transfected as indicated and cells were treated with MG132 for 6 hours. Ubiquitination of ΔNp73 was detected by immnoblotting with HA antibody after immunoprecipitation with anti-HA antibody. **(b)** 293T cells were transfected as indicated and cells were treated with MG132 for 6 hours. Ubiquitination of ΔNp73 was detected by immnoblotting with ubiquitin antibody after immunoprecipitation with Flag antibody. **(c)** 293T cells were transfected with indicated combination of plasmids. p73 polyubiquitination was detected by denaturing immunoprecipiation with anti-Flag antibody followed by detection with anti-HA antibody.

Supplementary Figure 4: (a) 293T cell lysate expressing SFB-WWP2, WWP1, HACE1 or E6AP was added to GST or GST-PPM1G immobilized on glutathione sepharose beads. The

interaction of E3 ligases with PPM1G was assessed by immunoblotting with Flag antibody. The expression of GST and GST-PPM1G was shown by coomasie staining. **(b)** 293T cells were transfected with PPM1G and increasing concentrations of WWP2, and their protein levels were detected by immunoblotting with Myc and Flag antibodies respectively. Actin was used as a loading control. **(c)** HeLa cells were transfected with either control siRNA or PPM1G siRNA. 24 hours later, cells were transfected with vector control, p73 alone or in combination with wild type WWP2. The percentage of apoptosis was determined by Propidium Iodide staining followed by sub-G1 peak analysis by using flow cytometry. Error bars indicate standard deviation (n=3), $P<0.01$; students *t-test.*

Supplementary Figure 5: (a) Cells were either left untreated or treated with cisplatin for 24 hours. The levels of WWP2, WWP1 and PPM1G were detected by immunoblotting with their respective antibodies. **(b)** HeLa cells were mock treated, cisplatin alone or together with CdCl2 and the percentage of apoptosis was determined by Propidium Iodide staining followed by sub-G1 peak analysis by using flow cytometry. Error bars indicate standard deviation (n=3), $P<0.01$; students *t-test.*
