# Supplementary material for: WWP2-WWP1 Ubiquitin Ligase Complex Coordinated by PPM1G Maintains the Balance between Cellular p73 and ΔNp73 Levels
Source: Mol Cell Biol. 2014 Oct;34(19):3754–64. doi: 10.1128/MCB.00101-14 (PMC4187731; doi:10.1128/MCB.00101-14)
Supplement: Supplemental material [file MCB.00101-14_zmb999100602so2.pdf]

Consolidated list of WWP2 associated proteins identified by Mass spectrometric analysis:

WW domain containing E3 ubiquitin protein ligase 2 (WWP2)

Ubiquitin specific peptidase 9, X-linked(USP9X)

Angiomotin(AMOT)

PYD (pyrin domain) containing 1(PYC)

methylcrotonoyl-CoA carboxylase 2 (beta) (MCCC2)

propionyl CoA carboxylase, beta polypeptide (PCCB)

uveal autoantigen with coiled-coil domains and ankyrin repeats (NUCL)

optic atrophy 6 (autosomal recessive) (ROA1)

RNA binding motif protein (RBM14)

nucleophosmin (nucleolar phosphoprotein B23, numatrin) (NPM)

DEAD (Asp-Glu-Ala-Asp) box helicase 17 (DDX17)

HLA-B associated transcript 2 (BAT2)

heterogeneous nuclear ribonucleoprotein K (HNRPK)

WW domain containing E3 ubiquitin protein ligase 1 (WWP1)

DEAH (Asp-Glu-Ala-His) box polypeptide 9 (DHX9)

angiomotin like 1 (AMOL1)

glucose-regulated protein, 78kDa (GRP78)

DEAD (Asp-Glu-Ala-Asp) box helicase 5 (DDX5)

GTPase activating protein (SH3 domain) binding protein 1 (G3BP1)

GTPase activating protein (SH3 domain) binding protein 2 (G3BP2)

WW domain binding protein 2 (WBP2)

itchy E3 ubiquitin protein ligase (ITCH)

fragile X mental retardation 1 (FMR1)

tumor protein p73 (p73)

heterogeneous nuclear ribonucleoprotein U-like 1 (HNRNPUL1)

ubiquitin specific peptidase 7 (USP7)

protein phosphatase, Mg2+/Mn2+ dependent, 1G (PPM1G)

polymerase (RNA) II (DNA directed) polypeptide C (POLR2C)

polymerase (RNA) II (DNA directed) polypeptide E (POLR2E)

eukaryotic translation elongation factor 2 (EEF2)

RNA polymerase II associated protein 2 (RPAP2)

eukaryotic translation initiation factor 4A1 (EIF4A1)

solute carrier family 25 (mitochondrial carrier; adenine nucleotide translocator), member 5 (SLC25A5)

pyruvate carboxylase (PC)

ubiquitin specific peptidase 15 (USP15)

family with sequence similarity 175, member B (FAM175B)

dehydrogenase/reductase (SDR family) member 7B (DHRS7B)

ATP synthase, H+ transporting, mitochondrial F1 complex, alpha subunit 1, cardiac muscle (ATP5A1)

Dishevelled-2 (DVL2)

proliferating cell nuclear antigen (PCNA)

nudix (nucleoside diphosphate linked moiety X)-type motif 21 (NUDT21)

WD repeat domain 5 (WDR5)

OTU domain containing 7B (OTUD7B)

glycogen synthase 1 (GYS1)

spastic paraplegia 20 (Troyer syndrome) (SPG20)

ubiquitin specific peptidase 24 (USP24)

5'-3' exoribonuclease 2 (XRN2)

cofilin 1 (CFL1)

trimethyllysine hydroxylase, epsilon (TMLHE)

GPN-loop GTPase 3 (GPN3)

GPN-loop GTPase 1 (GPN1)

RecQ protein-like 5 (RECQL5)

eukaryotic translation elongation factor 1 alpha 2 (EEF1A2)

Deleted in azoospermia-associated protein 1 (DAZAP1)

brain and reproductive organ-expressed (TNFRSF1A modulator) (BRE)

glutamate receptor, ionotropic, N-methyl D-aspartate-like 1A combined protein (GRINL1A)

protein tyrosine phosphatase, non-receptor type 14 (PTPN14)

heterogeneous nuclear ribonucleoprotein F (HNRNPF)

protein phosphatase 2, regulatory subunit B, alpha (PPP2R2A)

ADP-ribosylation factor 1 (ARF1)

lysosomal protein transmembrane 4 alpha (LAPTM4A)

chaperonin containing TCP1, subunit 2 (beta) (CCT2)

RNA polymerase II associated protein 3 (RPAP3)

histone cluster 1, H4j (HIST1H4J)

protein phosphatase 2, regulatory subunit A, alpha (PPP2R1A)

enolase 1, (alpha) (ENO1)

tyrosine 3-monooxygenase/tryptophan 5-monooxygenase activation protein, epsilon polypeptide (YWHAE)

protein arginine methyltransferase 5 (PRMT5)

H2B histone family, member Q (HIST2H2BE)

prolactin-induced protein (PIP)

polymerase (RNA) II (DNA directed) polypeptide D (POLR2D)

Silencer of death domains (BAG4)

PDZ and LIM domain 7 (PDLIM7)

transglutaminase 3 (TGM3)

eukaryotic translation elongation factor 1 gamma (EEF1G)

synaptotagmin binding, cytoplasmic RNA interacting protein (SYNCRIP)

lactotransferrin (LTF)

angiomotin like 1 (AMOTL1)

serine/threonine kinase receptor associated protein (STRAP)

guanine nucleotide binding protein (G protein), beta polypeptide 2-like 1 (GNB2L1)

Tongue cancer chemotherapy resistance-associated protein 1 (FAM168A)

eukaryotic translation initiation factor 4A3 (EIF4A3)

CDK5 regulatory subunit associated protein 1 (CDK5RAP1)

Cell cycle progression restoration gene 3 protein (DNAJA2)

protein kinase, AMP-activated, gamma 1 non-catalytic subunit (PRKAG1)

tyrosine 3-monooxygenase/tryptophan 5-monooxygenase activation protein, theta polypeptide (YWHAQ)

dishevelled-1 (DVL1)

plasminogen activator inhibitor 1 RNA binding protein (SERBP1)

DNA damage-regulated overexpressed in cancer 45 protein (OLA1)

DnaJ (Hsp40) homolog, subfamily A, member 1 (DNAJA1)

Cytosolic thyroid hormone-binding protein (PKM2)

dolichyl-phosphate mannosyltransferase polypeptide 1, catalytic subunit (DPM1)

phenylalanyl-tRNA synthetase, alpha subunit (FARSA)

coiled-coil domain-containing protein 85C (CCDC85C)

Multiple substrate lipid kinase (AGK)

BRCA1/BRCA2-containing complex, subunit 3 (BRCC3)

N-ras upstream gene protein (CSDE1)
