# Supplementary material for: WWP2-WWP1 Ubiquitin Ligase Complex Coordinated by PPM1G Maintains the Balance between Cellular p73 and ΔNp73 Levels
Source: Mol Cell Biol. 2014 Oct;34(19):3754–64. doi: 10.1128/MCB.00101-14 (PMC4187731; doi:10.1128/MCB.00101-14)

Supplementary Figure 1

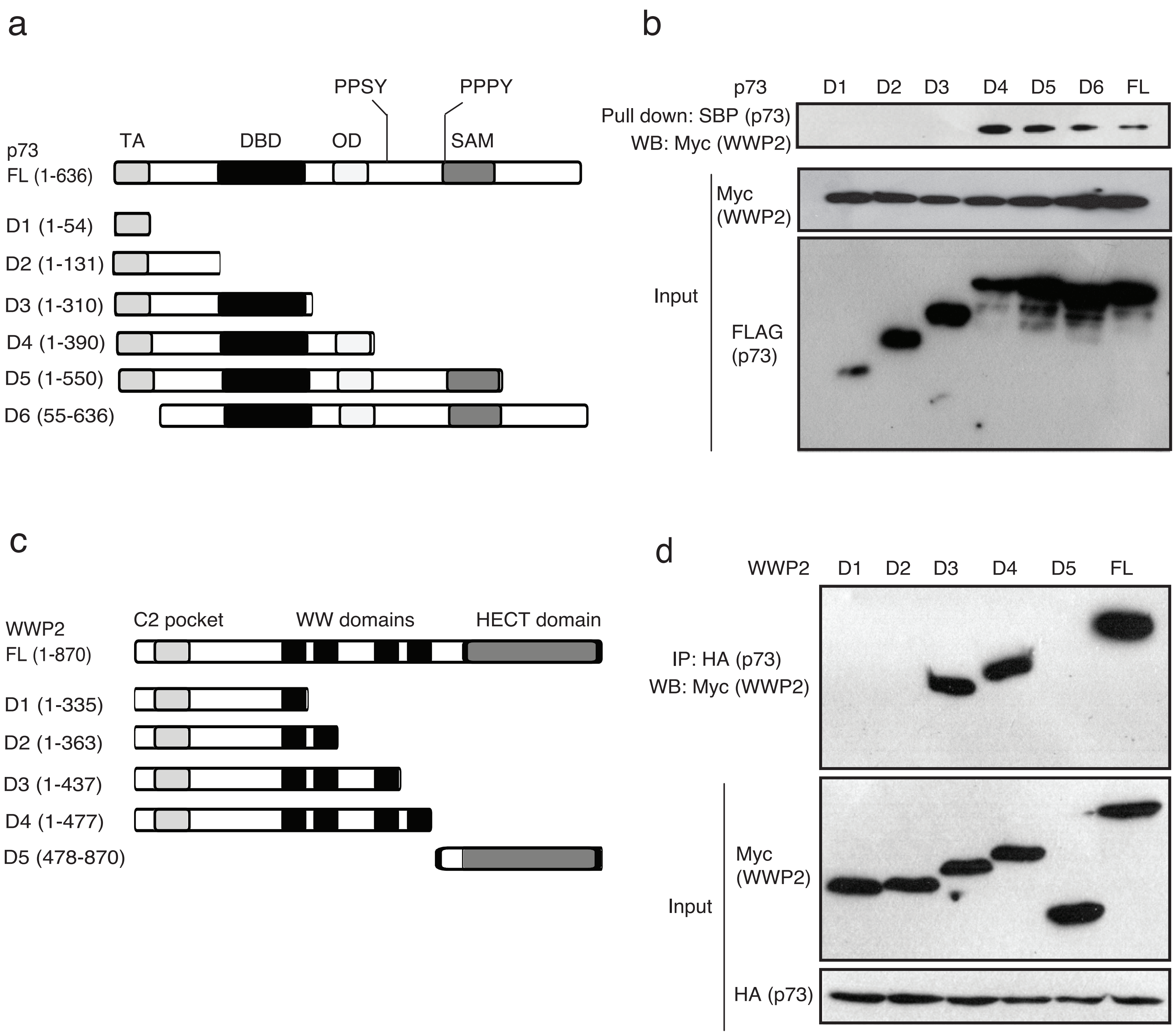

Supplementary Figure 2

a

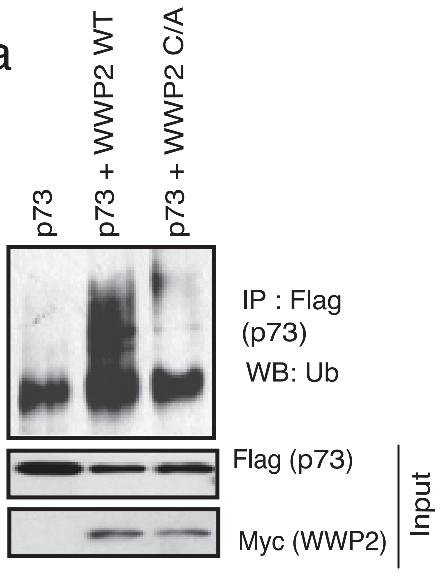

b

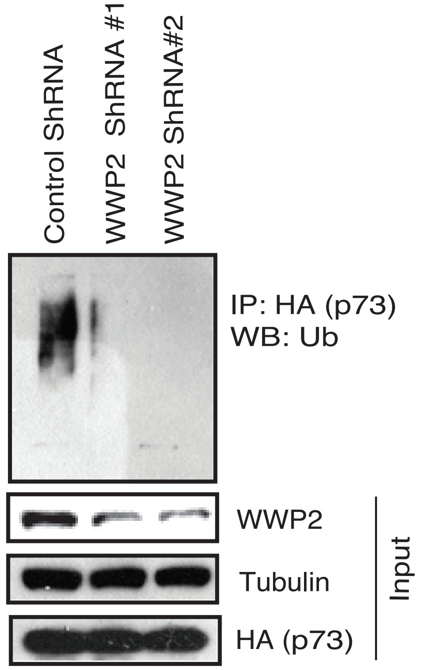

c

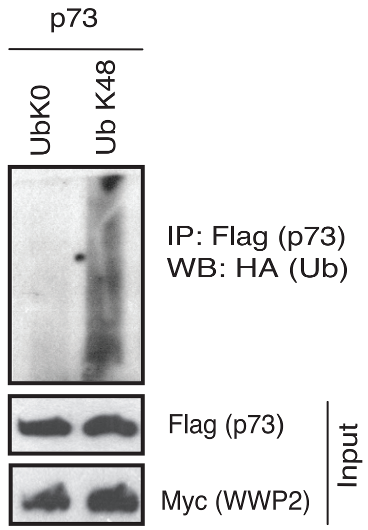

Supplementary Figure 3.

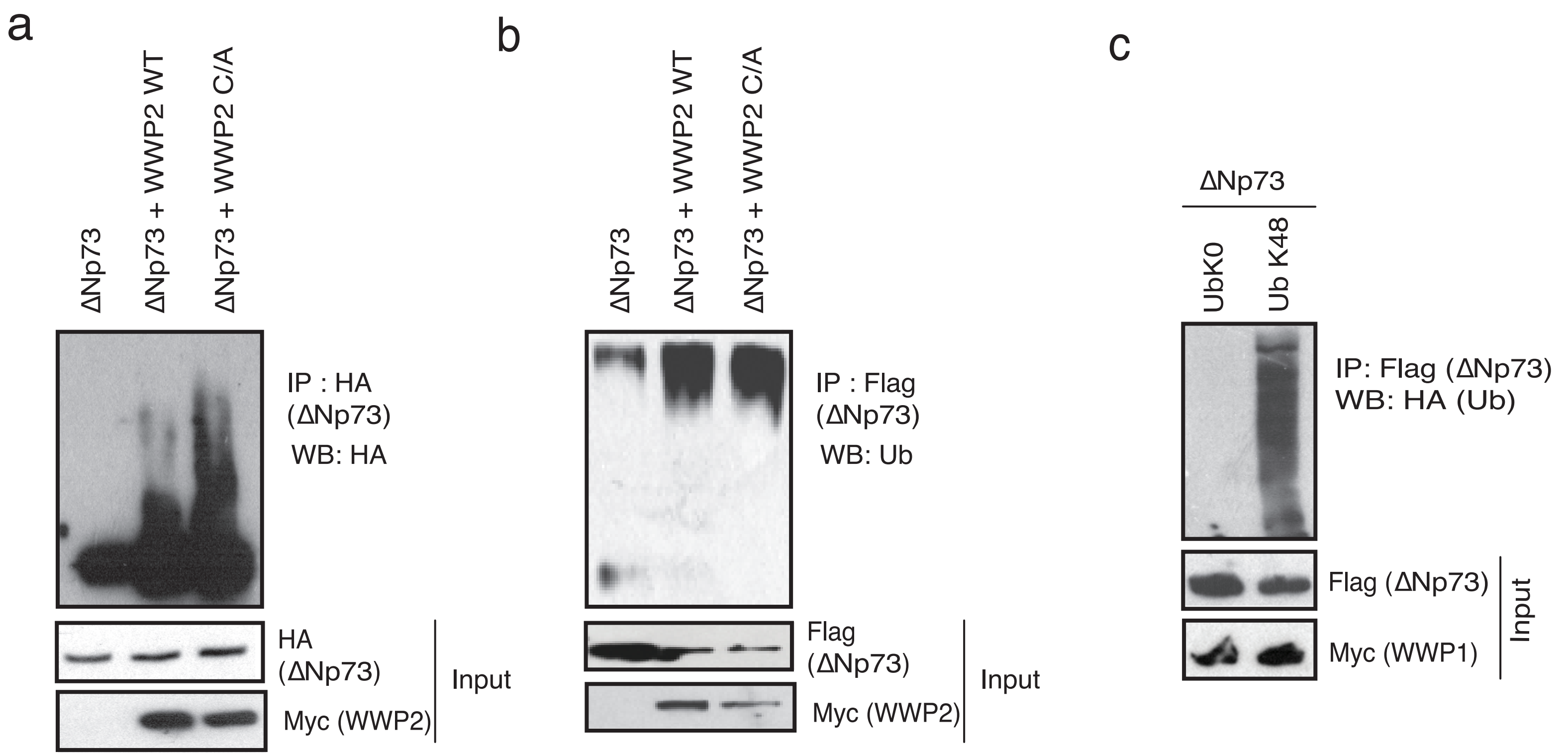

Supplementary Figure 4.

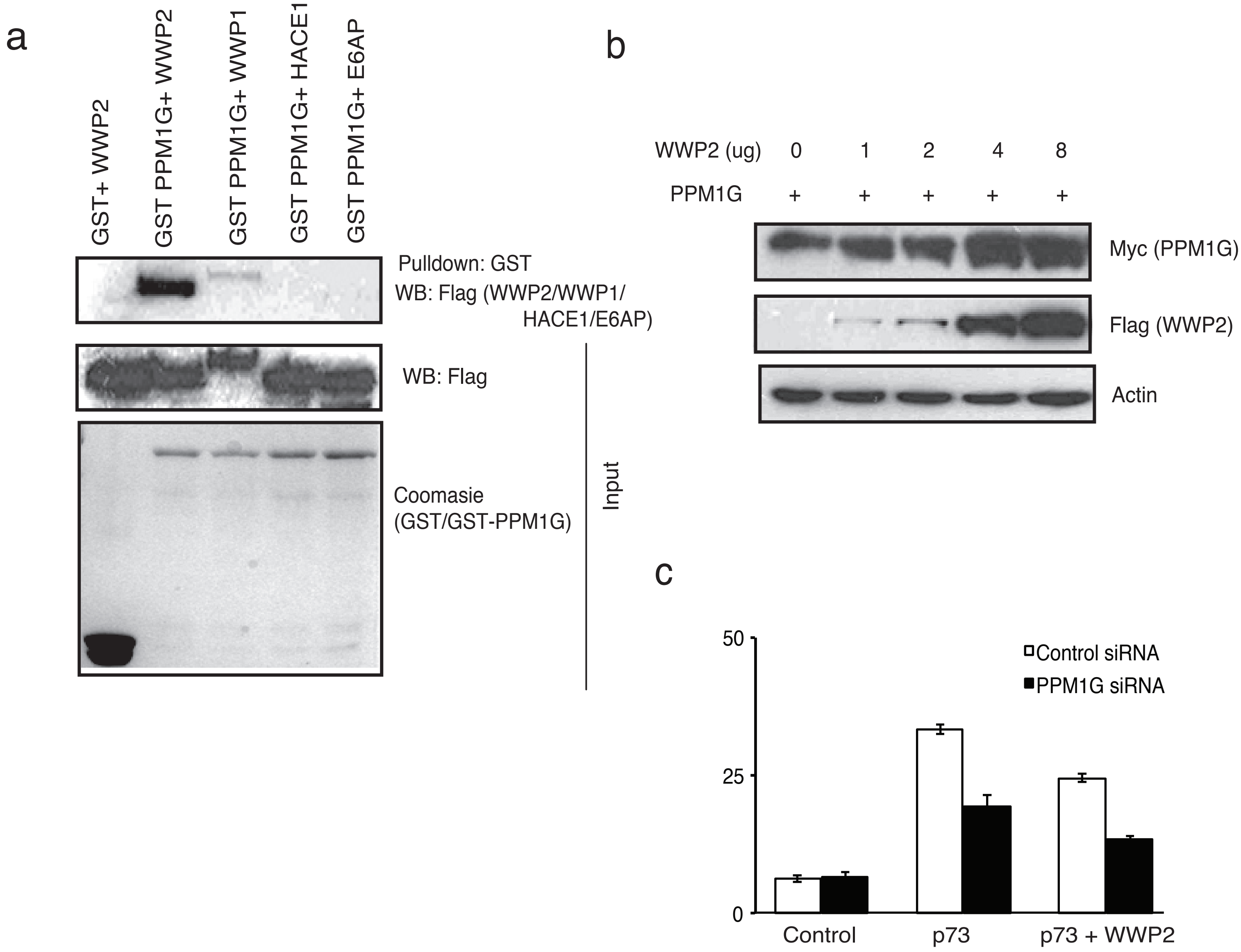

Supplementary Figure 5.

a

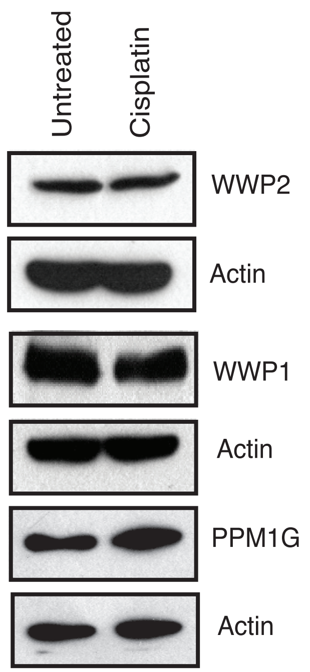

b

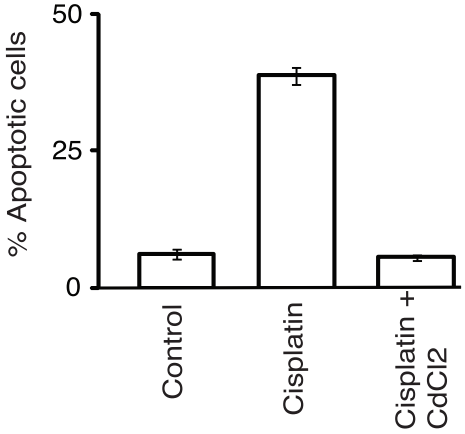

Supplement: Supplemental material [file MCB.00101-14_zmb999100602so3.pdf]
